# Facilitators and barriers to recruitment and retention in a feasibility trial of encapsulated faecal microbiota transplant to eradicate carriage of antibiotic-resistant bacteria at an academic hospital in central London: a nested qualitative study

**DOI:** 10.1136/bmjopen-2025-104783

**Published:** 2025-11-19

**Authors:** Blair Merrick, Désirée Prossomariti, Michael Kertanegara, David Wyatt, Simon Goldenberg

**Affiliations:** 1 Guy's and St Thomas' NHS Foundation Trust and King’s College, London, Centre for Clinical Infection & Diagnostics Research, London, UK; 2 Department of Population Health Sciences, King’s College London, London, UK

**Keywords:** QUALITATIVE RESEARCH, Feasibility Studies, Gastrointestinal Microbiome, Microbiota, Patient Participation

## Abstract

**Objectives:**

This nested qualitative study (NQS) aimed to identify facilitators and barriers to the delivery of a substantive randomised controlled trial investigating the eradication of gastrointestinal tract carriage of antibiotic-resistant organisms using encapsulated faecal microbiota transplant (FMT).

**Design:**

NQS within a participant-blinded, randomised, placebo-controlled, single-centre, feasibility trial (RCT)—Feasibility of ERadicating gastrointestinal carriage of Antibiotic-Resistant Organisms (FERARO) (ISRCTN reg. no. 34 467 677)—with data collected via focus groups and analysed using thematic analysis.

**Setting:**

RCT participants were recruited from a large academic tertiary referral hospital in central London. Focus groups were held at the hospital or via videoconferencing for those unable to travel.

**Participants:**

This study included 13 FERARO study participants across two focus groups. 11 participants were under RCT follow-up and unaware of their treatment allocation, two participants had completed 6-month follow-up and knew whether they had received FMT or matched placebo. Additional data were opportunistically collected on reasons for declining RCT participation.

**Results:**

Participants found FMT to be an acceptable and holistic management strategy and noted positive impacts from RCT participation including enhanced personal health awareness and valuable support from the research team. The time and travel commitment presented the most substantial barrier to RCT participation. Many participants were motivated by a desire to give something back to the UK National Health Service and/or research. Patients’ current health status also influenced the decision-making process, and, while infrequently cited, the COVID-19 pandemic added extra complexity likely impacting individuals’ willingness to participate.

**Conclusions:**

While FMT is generally acceptable to participants, logistical barriers such as the time and travel commitment associated with RCT participation need consideration. Effective communication, personal connections and participant education on antimicrobial resistance are likely to be crucial for enhancing recruitment and retention in future trials.

**Trial registration number:**

ISRCTN registration number 34 467 677 and EudraCT number 2019-001618-41.

**STRENGTHS AND LIMITATIONS OF THIS STUDY**

- *Innovative approach:* the Feasibility of ERadicating gastrointestinal carriage of Antibiotic-Resistant Organisms (FERARO) trial combined qualitative (nested qualitative study) and quantitative (randomised controlled trial (RCT)) methodologies, providing a comprehensive understanding of patient experiences and feasibility of conducting a substantive clinical trial.
- *Participant engagement:* focus groups offered in-depth insights into participant perspectives; these will help inform the design and implementation of a substantive RCT investigating multidrug-resistant organism gastrointestinal tract decolonisation, as well as future faecal microbiota transplant trials.
- *Real-world setting:* conducted in a large academic centre, the FERARO trial findings are grounded in a pragmatic healthcare environment, enhancing their relevance to real-world settings.
- *Sample size and scope:* the study included a small sample size and was conducted amidst the COVID-19 pandemic, which may limit the generalisability to a broader population.
- *Exclusion of non-participants:* patients who declined to participate in the FERARO trial did not participate in focus groups. Although we did attempt to collect reasons for non-participation, we potentially missed critical insights into their perceived barriers to RCT participation.

## Introduction

 Antimicrobial resistance (AMR) is a major global threat.^1^ The greatest risks arise from organisms with certain resistance mechanisms, particularly extended-spectrum beta-lactamase (ESBL-E) producing and carbapenem-resistant Enterobacterales (CRE),^2^ for which novel treatments are a critical priority. The human gastrointestinal tract (GIT) is a major reservoir of multidrug-resistant organisms (MDROs). Colonisation often precedes infection; while some individuals decolonise spontaneously, no established eradication strategy exists for persistent carriers.^3^,^5^

Faecal microbiota transplant (FMT) involves transferring minimally manipulated screened donor faeces to restore or ameliorate a recipient’s gut microbiota. It is effective in the management of *Clostridioides difficile* infection (CDI)^6^ and recommended in several national and international guidelines.^7^,^11^ FMT may also help manage AMR. Some studies in recurrent CDI report reduced GIT MDRO carriage and antibiotic resistance genes,^12 13^ though randomised controlled trials (RCTs) show mixed results.^14 15^ While FMT and closely related stool-based products are generally acceptable for CDI, little is known about participants’ experience of taking FMT, delivery route preferences or acceptability beyond hypothetical scenarios, with further research recommended.^16^,^18^

The Feasibility of ERadicating gastrointestinal carriage of Antibiotic-Resistant Organisms (FERARO trial) was a randomised (1:1), controlled, participant-blinded, single-centre, feasibility trial (RCT).^19 20^ Participants needed to attend several in-person hospital-based appointments; trial screening, baseline data collection and three FMT dosing visits were conducted over consecutive days prior to a 6-month follow-up period with three remote and one in-person visit. 44 participants colonised with an ESBL-E or CRE were randomised to receive lyophilised, encapsulated FMT or matched placebo. 41 participants went on to receive Investigational Medicinal Product (IMP) and 38 participants completed follow-up. The primary outcome was the participant consent rate and based on a priori defined criteria; this deemed a substantive trial would be feasible with protocol modifications.^20^

Here, we report on a nested qualitative study (NQS) within the FERARO RCT designed to capture additional data that were not specifically codified for in the protocol. The objectives of the NQS included identifying facilitators and barriers to delivering a substantive RCT, identifying ways to increase recruitment and retention and broadening participation to such a study. It also set out to improve the understanding of how and why participants may join trials, their experiences of participation and exploring views on the acceptability of RCT design and encapsulated FMT.

## Methods

### Methodological guidelines

The NQS is reported in accordance with the consolidated criteria for reporting qualitative research (online supplemental file 1).

### Design of the NQS

24 participants undergoing or who recently (within 6 months) completed RCT follow-up were invited to attend one of two focus groups held on Monday 7 February 2022 and Tuesday 6 December 2022 at St Thomas’ hospital, London, UK. 13 (54%) accepted the invitation and provided written consent. Of these, 11 were between the 3-month and 6-month follow-up visits, and two had completed study follow-up and were aware of their treatment allocation. Participants averaged 66 years of age (IQR 59–78 years), were predominantly female (69%, 9 participants) and of white ethnicity (92%, 12 participants). Invitations were sent out via post to all (19) participants between the penultimate and final FERARO trial visits at the time of each focus group based on a desire for both recent and extensive trial experience. An additional five participants who had completed all follow-up visits were invited based on the anticipated consent rate (≈50%) and the desired focus group size (4–8 participants). 12 participants attended (seven to the first focus group and five to the second) in person, and one participant requested to attend the first focus group via videoconferencing as they lived >1 hour from the hospital. It was not deemed feasible to have individuals who did not consent to participate in the RCT attend either focus group. However, reasons for non-participation were collected opportunistically; these data also formed part of the analysis.

The focus groups were facilitated by the Chief Investigator and Consultant Microbiologist, SG (MBBS, MD(Res)), of the FERARO trial who had not previously met the participants. Other members of the research team were present (BM and DP at both, MK at second focus group). A social science researcher and senior research fellow, DW (PhD), with experience in focus group methods was also present at both focus groups.

Each focus group was structured around a guidance document (see online supplemental file 2). The focus groups began with a presentation by SG which provided some background on FMT, the RCT and this study. Participants were then asked to introduce themselves to the group to try and create an environment in which individuals felt comfortable sharing their experiences and opinions. Participants were then prompted with open questions including reasons for participation, health benefits they encountered through participation, what went well and not so well during their participation and their perceptions of FMT. Each focus group lasted 2 hours and was audiovisually recorded and auto-transcribed using Microsoft Teams (V.10.4.7 and 16.2.8). Each transcript was verified for accuracy against the original audiovisual recording before anonymisation. As this was an exploratory study, the aim was to gain wide-ranging and textured data on participants’ understandings and experiences of FMT and the FERARO trial. Data saturation was not the aim nor viable with a potential sample of 44 (all randomised trial participants).

### NQS analysis

Using thematic analysis, transcripts were coded for a descriptive account independently by both BM and MK using NVivo (release V.14.23.0).^21 22^ Any disagreements were discussed with refinements until agreement was reached. Themes were derived from the coded data by BM and discussed and refined with DW. Participants were not asked to provide feedback on the findings.

### Patient and public involvement

Patients and the public identified AMR as a research priority. A patient representative was appointed to the trial steering committee of the FERARO trial, and as the primary objective was to determine the feasibility of a substantive trial, it was decided to incorporate focus groups to understand participant experiences focusing on acceptability, barriers to participation and improvements that could be made to any resulting trial.

## Results

### Central themes

**Figure 1:**
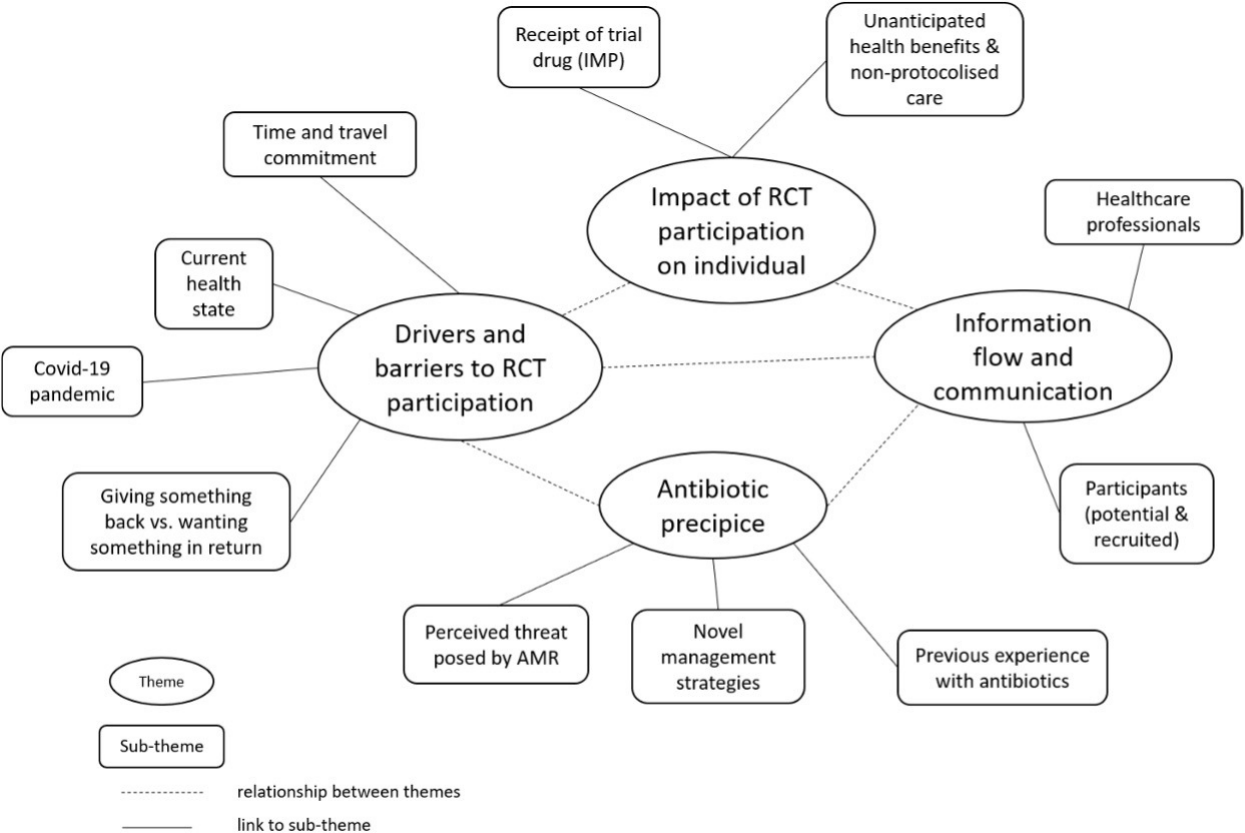
Map of central and subthemes and relationships between them derived from thematic analysis of data. AMR, antimicrobial resistance; RCT, randomised controlled trial. IMP, Investigational Medicinal Product.

Below are four themes, each with subthemes, that were central to the accounts of focus group participants and individuals who did not consent to participate in the FERARO trial. The links between the themes and subthemes are represented pictorially in figure 1.

- Drivers and barriers to RCT participation:
  1. Time and travel commitment.
  2. An individual’s current health state.
  3. Giving something back versus wanting something in return.
  4. COVID-19 pandemic.
- Impact of RCT participation on an individual:
  1. Receipt of FMT.
  2. Unanticipated health benefits and non-protocolised care.
- Information flow and communication between the research team and
  1. Potential and recruited participants or
  2. Other healthcare professionals (HCPs).
- The antibiotic precipice:
  1. Prior experience of antibiotic therapy.
  2. The perceived threat posed by AMR.
  3. Novel management strategies.

#### Drivers and barriers to RCT participation

This first theme focuses on the reasons to participate (or not) in the FERARO trial. Four main drivers and barriers to participation were identified in the analysis.

##### The time and travel commitment

Time commitment was the most common reason for non-participation, with unwillingness to travel to the hospital also frequently cited (table 1). It was anticipated most participants would be recruited while an inpatient and undertake most of the face-to-face elements of the trial prior to discharge. However, this occurred for a single participant only and meant most needed to attend as outpatients for IMP dosing over three consecutive days. Even among those who consented, the time and travel commitment were identified as barriers, “I was only happy because I had so many medical appointments…I wouldn’t have wanted to come back three days running, just for tiredness. I mean my health is not that great either. I think it is a problem. It is a barrier.” Or, if not directly identified as a barrier, that it might present an impediment to some, “I was surprised about how many visits there were, which was fine as I’d agreed to do it. I imagine that somebody might think well, that seems quite a lot”.

**Table 1:** Reasons for non-participation in the FERARO trial

Main reason cited for declining participation	No.	%
Time commitment/inconvenient	32	30.5
Not interested in research	17	16.2
Concurrent medical problems	15	14.3
Unable/unwilling to travel	12	11.4
Moving away	8	7.6
Study concept/taking FMT	8	7.6
External influence (family/friends/clinician)	5	4.8
Safety concerns	3	2.9
No perceived benefit from participation	3	2.9
Lack of payment	2	1.9
**Total**	**105**	**100**

FERARO, Feasibility of ERadicating gastrointestinal carriage of Antibiotic-Resistant Organisms; FMT, faecal microbiota transplant.

Three out of four follow-up visits were conducted remotely, with stool samples returned via pre-paid Royal Mail Safebox.^23^ However, the Safebox (purchased in early 2020) had issues; for example, participants described them as, “very difficult to close” and highlighted, “the container doesn’t fit in the post-box, so you’ve actually got to physically take it to an open Post Office and give it in over the counter”. This impacted on individuals’ social lives, “I had an occasion where I was meeting somebody and I was late when I was going to the pub and I had it with me, and it wouldn’t fit in, so I then had to sit in the pub with it for a few hours”, and worse still, “my husband had to take it to the Post Office for me and then he got the third degree about what’s inside it”. This problem was raised with Royal Mail, leading to a product redesign in 2022 and improved staff training should boxes be dropped off at the counter.

##### Current health state

Motivation to participate often came from not being well, “two motivations, to get healthier…”; concern about deterioration, “it’s not just about how sick you are to force yourself to do something, it is also how worried you are that you can get sicker” or unresolved medical issues for which there was no treatment solution, “I have been at Guy’s (hospital) for over three years with an infection that won’t go away”; another said, “I had a few urinary tract infections quite close together, and I was quite frustrated by that, because it didn’t feel right to have this thing going on”. Several individuals declined as they currently felt well, “I don’t have any problems at the moment,” or were “happy with my current treatment strategy”.

At the other end of the spectrum, some declined due to competing health issues, “too many other medical issues” or they would “consider (the) trial when less busy with other health issues” or that they would not be able to complete trial visits due to “severe back pain”, “bad anxiety” or being “needle phobic”. Since risk of MDRO colonisation increases with comorbidities and healthcare contact,^24 25^ those most likely to meet eligibility criteria were often the least able to undertake trial activities.

##### To give something back or want something in return?

Participants often talked of wanting to give back, whether from personal experience, “I had fantastic treatment, and I wanted to give something back”, or that of relatives or friends, “because of my brother who had cancer…he’s had radiotherapy, chemo, he’s had everything, but he was also on two trials…so that was the reason I came and thought I’d give something back as well”. Others mentioned awareness of research challenges, “I used to share a house with a doctor…he always had trouble recruiting people”. For some, research participation reached an even greater level of personal responsibility, “I think one has an obligation, a moral obligation, to participate in something like this. Life is finite anyway, so that’s about it”.

Some declined participation as they felt the trial offered nothing in return, such as financial payment, only reasonable travel expenses were reimbursed. Participants recognised the precedent for financial incentives in research studies, “I do the ONS [Office for National Statistics] COVID-19 study…then I found out they’re sending me, you know, emails with vouchers, and I thought this is good”; others opposed payment on principle but acknowledged it could help, “I don’t agree with getting paid either, but I can see the incentive might make a difference”. Alternative suggestions included gut microbiome results, “for us patients to know since it’s such a hot issue at the moment, the microbiodome, what our status is”. There were discussions in both focus groups about charity donations, “goodie bags” and other incentives, but no consensus as to what was reasonable was reached.

##### COVID-19 pandemic

FERARO recruitment coincided with the COVID-19 pandemic, opening after the first wave in September 2020, pausing as the second started in late 2020 and restarting in Spring 2021. Many participants were clinically extremely vulnerable and advised to isolate. While COVID-19 was rarely cited as a reason for non-participation (table 1) or mentioned as a barrier or motivator for those who did participate, “didn’t think about it”, “didn’t affect me” and may have even generated a “great deal more interest” in infection management, its impact cannot be ignored, “I think there’s been fatigue among people about anything medical like, yeah, not another thing”; “during COVID…you get incentivised to get the vaccine, do this, do that. All these, you know, restrictions and not restrictions, rules over rules, so some people might have perceived this (participation) as another thing that is forced onto you”. Also, “I think our timing has been a bit unfortunate. I think Covid has got in the way”. This suggests that the COVID-19 pandemic is likely to have been a contributory factor in many individuals’ decision-making process.

### Impact of FERARO participation on the individual

RCT participation involves receiving an intervention which may have limited safety and efficacy data. While many hope for predefined health benefits, unanticipated health gains or non-protocolised care may also arise.

#### Receipt of FMT

The dichotomy between faeces being harmful, “often we think faeces, careful, you need to wash your hands”, as well as helpful “you get something healthy from someone else”, was highlighted. None expressed concerns regarding receiving FMT and trusted its safety, “you wouldn’t give me something dangerous”. This was reinforced by the willingness and even desire for most participants to take the study IMP at home, “it would really help if people could just take it (the IMP) at their home”, suggesting they felt confident taking FMT outside of a healthcare setting. One remarked, “I was personally fine with that (taking FMT), because I understood the wider context (rationale for microbiome restoration therapy)”. The participants did recognise that they were a group that had consented to the study, and wider public opinions may differ. They were concerned that the term, ‘faecal microbiota transplant’ may put individuals off, “do you think that the name, the terminology scares people”, and could benefit from rebranding “lose the word faecal in it. Bio-something, bio microbiota, or biodome? People, now, are beginning to get aware of something by gut biodome”. Branding was seen as an acceptable technique to improve appeal, and not an underhand tactic to mask what FMT contains. Although product nature was given as a reason for declining to participate, it was less frequent than other reasons discussed within the first central theme (table 1).

The capsule formulation was generally acceptable, with no issues swallowing it or concerns about smell or taste. However, the colour (bright brown/orange) did cause some concern: “slightly alarmed at the colour, I kind of wanted them to be white”, “is that [the colour] a joke?”

#### Unanticipated health benefits and non-protocolised care

Before unblinding, not all participants would be drawn on whether they had experienced health benefits. Some did not identify benefits, aside from their interaction with the trial team, “I don’t think it has made any difference to me…It’s lovely to meet Blair [trial coordinator] obviously, but I don’t think there’s been any difference really, personally”. Others noted improvements in their health, though not necessarily because of the trial or intervention, “I’m a bit less tired now, in fact, far less tired than I was, even a month ago. So, don’t know if it is down to, if it is all in the head, or all in the gut”, or recovery over time, “At the beginning, I really couldn’t walk…but over the course of the study, I’ve gotten much better”. Some described fewer infections, “since I’ve started, I’ve not had any bacterial infections, so I’ve not had to take any antibiotics”—a phenomenon reported in other FMT studies, although not directly assessed in FERARO.

For those with ongoing infections, support from the trial team was highly valued, “I sent Blair a message. Oh, he was like just sure, send a sample here and we’ll test it, and I thought that was actually really helpful…that was all really, really, fabulous extra care.” Participants highlighted psychological benefits, “I was encouraged for the interest that you were taking in the patients, because I’m so removed from the GP, that is was psychologically very good for me.” Another remarked, “Oh, admittedly, it was very good for me. I think this is an absolutely key point”, and another, “yes, that’s a big positive of being in the trial”. There were also incidental physical benefits—a participant was identified as having previously undiagnosed iron deficiency anaemia and referred on for investigation.

### Information flow

Potential FERARO participants were initially contacted by post. When it became apparent that correspondence was not reaching them, a follow-up phone call was initiated to check receipt of the participant information sheet (PIS), a document approved by the Research Ethics Committee (REC) during trial set-up, which was resent where needed. Issues with the postal service (Royal Mail) were probably related to a combination of COVID-19 and workforce strikes. Communication with participants forms the first subtheme. Hospital departments were briefed and consented to support recruitment, while general practitioners (GPs) were contacted when one of their patients was recruited. Communication between HCPs is the subject of the second subtheme.

#### Participants

The PIS suited some, but not all approached participants. Some found it too ‘official’, while others were concerned it was overly detailed and people might get “overwhelmed by it” or few would read it “maybe 20%”. One suggested, “if it could be one sheet and if people want to read more, there could be this [FERARO PIS] what you produce as well”. The concept of tailoring messaging to different groups, using different channels such as e-mail or video, was thought to be acceptable; “there’s nothing wrong, I think, personally, ethically in having differently tailored messages for different groups”; direct verbal communication with the team and rapport was deemed important, “He (BM) is very convincing…it comes down to personal relationships, one-to-one.” Some thought it acceptable to “oversell it a bit” and that positive messaging might improve uptake, “I think it started off by saying you’ve been identified as having bad bacteria, perhaps turning it around and saying, are you aware there’s a global problem in antibiotic resistance?” and “You could help this whole process happen better and faster if you take part in the trial”. Some thought that there were insufficient facts and figures presented to underline the threat of AMR, “I think maybe if you show some statistics at the very beginning… I was really quite shocked at the extent and that it might be more of a necessity for people to come and take part”.

Some participants were prepared, “my GP had phoned me 2 weeks before”; for others, it came out of the blue, “I was a bit surprised when I got contacted…you know I don’t mind, but I wasn’t expecting it” and that the approach might have caused distress, “I imagine some people may be quite alarmed”. As most participants were recruited from the community, this could reflect limited time for GPs to discuss the specifics of their case, and/or the increasing normality of MDRO infections.

#### Healthcare professionals

One participant was invited directly by their treating physician. This was deemed a “good thing” and suggests some functionality in internal hospital communication channels. Others not approached in this way would have found it preferable, “I think it might be a good idea if it came via your doctor”, reflecting a missed opportunity for individuals with regular hospital contact. Although participants were given alert cards to show HCPs, these rarely prompted interest, and direct care teams barely contacted the research team during the study.

Some declined after being advised against participation by non-Guy’s and St Thomas’ NHS Foundation Trust (GSTT) doctors who had not contacted the research team and were thus unlikely to have all the available information regarding the trial on which to base their advice.

### The antibiotic precipice

Without changes to antimicrobial use and infection control practices, a postantibiotic era looms, and despite awareness campaigns, misconceptions surrounding AMR persist.^26^ The first subtheme considers participants’ understanding of AMR. Participants’ experiences with antibiotics are captured in the second subtheme (all had recent exposure for treatment of MDRO infections). Previous negative experiences with antibiotics, and a recognition of their inherent limitations coupled with the growing threat of AMR, led to the participants expressing an interest in novel management strategies (third subtheme).

#### The perceived threat posed by AMR

Most participants were aware of AMR, but possibly not its extent, “I was really surprised to find it was so prevalent…I was severely shocked to find out how widespread it is”, where it was coming from, “I was amazed about the amount of it coming through the food chain” or being personally impacted came as a revelation to many, “I was quite surprised, I don’t think I’ve taken antibiotics very often at all”. There was, at times, confusion regarding what AMR was, specifically that resistance is a feature of bacteria, rather than it being the individual who becomes resistant to antibiotics.

Participants had not worried about AMR until personally affected “because it wasn’t affecting me personally”, but many recognised everyone had a role to play in managing it. There was a recognition that one needed to move away from a “time you went to the doctor and if you didn’t come away with a prescription [for antibiotics] you felt that you weren’t listened to”. They felt of themselves as informed about the limitations of antibiotics, but that others were not, “they [the uninformed] demand antibiotics as a quick fix, and you know, they’re given antibiotics and after a while, when they do need the antibiotics, they would have grown resistant”.

#### Prior experience of antibiotic therapy

Many participants, or their family members, had negative experiences with antimicrobials due to allergies, “I have to be careful anyway because I am allergic to penicillin, but a lot of the antibiotics I have been on have given me rashes and all sorts”, or intolerances, “I also have a problem with antibiotics generally”. Some avoided them unless essential, “not a fan of antibiotics really as a general rule, unless they are absolutely necessary”, and described a “vicious circle, you get more antibiotics, then you get more bacteria that are resistant and so on”. Frustrations included ineffective empirical antibiotics, “when you are given an antibiotic by your GP, very often it is kind of potluck, it’s like we are going to try you on those and if it doesn’t work then come back next week”.

#### Novel management strategies

Participants expressed curiosity, “I was just intrigued”; in whether FMT worked. Despite regulation as a medicinal product in the UK,^27^ many participants saw FMT as ‘alternative’, though not complementary medicine per se, “I don’t mean it in the sense of complementary medicine. I mean literally alternative ways of coping with diseases and conditions in modern society that don’t just rely on drugs”. Some described it as “natural” despite knowing it had been “tinkered with” during manufacture and a holistic treatment, “perhaps we’re learning a bit more that our own bodies could do a lot of healing as well, and that to encourage that process might be another way of looking at diseases”. This contrasted with more “traditional medicine” which can “lose the human body, the human organism, in the whole process”. There was broad appeal in using/repurposing nature. One participant recounted an article about a bivalve that produced an antibacterial compound, a “naturally produced antibiotic” and an optimism that “science might find another way of coping with that problem [AMR]”.

## Discussion

This NQS analysed the experiences and opinions of participants recruited to the FERARO trial, which assessed the feasibility of eradicating GIT carriage of antibiotic-resistant organisms using lyophilised encapsulated FMT. However, findings are likely to provide helpful insight into the design and delivery of FMT research studies more broadly.

Contrary to expectations, the ‘ick’ factor was not a major barrier to recruitment aligning with FMT given for other indications.^28^,^30^ NQS participants found FMT to be an acceptable and holistic treatment and adhered to dosing schedules, even when the alternative management strategy is to do nothing. As with other therapies associated with an ‘ick’ factor, for example, larval (maggot) therapy for non-healing/infected wounds, adequate explanation and rationale help individuals overcome the potentially unappealing nature of treatment.^31^ Physician reluctance remains a potential barrier that will be best addressed through greater awareness and evidence dissemination.^29^ Encapsulated FMT acceptability could be improved by design changes such as using white/colourless capsules consistent with previous work,^32^ provided blinding is ensured and there is no other impact on delivery. With the regulatory approval of stool-based products has come brand names including Rebyota^33^ and Vowst.^34^ NQS participants felt moving away from using the term faecal or FMT, for example, to ‘microbiome restoration therapy’, would enhance patient acceptability without obscuring the treatment origins.

Even among those engaged in AMR research, the scale of the issue was not fully appreciated, with many participants surprised at being personally impacted. They felt broader awareness could boost future trial participation. While interest in AMR has grown since the first *Lancet* report,^35^ it is not regularly featured in mainstream media. Unlike COVID-19, AMR is a chronic risk and lacks a clear crisis point,^36^ making it harder to capture attention, and a sustained effort is required to raise its profile.

The COVID-19 pandemic shifted research priorities in 2020,^37^ causing study pauses and reluctance to participate, although FERARO’s day-to-day running was largely unaffected. The trial co-ordinator’s (BM) commitment and enthusiasm supported trial activity adherence and was viewed positively in the NQS. Several participants described the impact of trial participation in terms of increased interest in their own health and medical problems. While difficult to objectify, this benefit has been noted in other similar trials.^28^ Personal motivations—whether for researchers or participants—are key drivers of engagement and should be fostered to maximise the success of future research projects.

Despite patient representative input, many commented that the PIS was unfit for purpose—too detailed, lacking detail or poorly delivered. A single written document is unlikely to serve all participants’ needs, particularly for larger substantive trials. Exploring alternative or personalised formats and collecting feedback and refining could help address this communication issue. There was an appetite for videos, digital material and face-to-face interaction over and above an impersonal generic document from participants, though REC approval will ultimately limit flexibility.

Only one participant was recruited and dosed as an inpatient, with eligibility often limited by competing medical issues or practical constraints. Outpatient recruitment was hindered by time and travel demands, suggesting that reducing inperson visits and community FMT dosing (supported by unpublished product stability data) would likely improve recruitment. Simplifying sample return will also be key. Recruitment was further limited by low engagement of direct care team HCPs; in a large, overstretched health system, expecting HCPs to have a working knowledge of all research studies and to consider eligibility of their patients is unrealistic. Automated tools that identify and flag potential participants to HCPs are likely to bolster recruitment.^38^

### Limitations

The NQS did not include patients who declined FERARO trial participation, so some barriers may have been missed. Findings are based on a small sample of individuals at a single centre and may not be representative of the broader patient population. In addition, two participants knew their treatment allocation, potentially impacting on their responses, and the study was conducted during the COVID-19 pandemic, which could limit the wider generalisability of findings. Finally, the NQS relied on self-reported data and was not independently run. Participants’ responses may have been influenced by this, for example, saying things to please the research team or providing socially desirable responses.

## Conclusions

The NQS within the FERARO trial provided valuable insight into the facilitators and barriers to participating in FMT research. While participants found FMT generally acceptable and holistic treatment for the eradication of MDRO colonisation, the time commitment and needing to travel for multiple study visits was a major barrier to the FERARO trial participation. Additionally, IMP acceptability could potentially be improved through rebranding and repackaging. The study highlighted the importance of a personal connection, finding and fostering personal motivation and effective communication in recruiting and retaining participants. Future trials could benefit from addressing logistical challenges, streamlining participant identification and in-person study visits and enhancing participant outreach and education on AMR. Despite limitations, the study offers guidance for designing more effective and inclusive FMT trials, including a substantive trial investigating the eradication of GIT carriage of MDROs.

## Supplementary material

10.1136/bmjopen-2025-104783 online supplemental file 1

10.1136/bmjopen-2025-104783 online supplemental file 2

## Data Availability

All data relevant to the study are included in the article or uploaded as supplementary information.
