# Supplementary material for: Facilitators and barriers to recruitment and retention in a feasibility trial of encapsulated faecal microbiota transplant to eradicate carriage of antibiotic-resistant bacteria at an academic hospital in central London: a nested qualitative study
Source: BMJ Open. 2025 Nov 19;15(11):e104783. doi: 10.1136/bmjopen-2025-104783 (PMC12636872; doi:10.1136/bmjopen-2025-104783)
Supplement: online supplemental file 1 [file bmjopen-15-11-s001.docx]

**Consolidated criteria for reporting qualitative studies (COREQ): 32-item checklist**

| **No. Item** | **Guide questions/description** | **Reported on Page #** |
| --- | --- | --- |
| **Domain 1: Research team and reﬂexivity** |  |  |
| *Personal Characteristics* |  |  |
| 1. Inter viewer/facilitator | Which author/s conducted the interview or focus group? | 2, SG and DW |
| 2. Credentials | What were the researcher’s credentials? E.g. PhD, MD | SG – MBBS, MD(Res), DW – PhD, 2 |
| 3. Occupation | What was their occupation at the time of the study? | SG – consultant microbiologist and chief investigator of FERARO trial, DW – Senior Research Fellow 2 |
| 4. Gender | Was the researcher male or female? | Both male, 1 |
| 5. Experience and training | What experience or training did the researcher have? | SG had no prior experience of focus group methods, DW has extensive experience, 2 |
| *Relationship with participants* |  |  |
| 6. Relationship established | Was a relationship established prior to study commencement? | Neither SG nor DW had met the participants prior to the focus group, 2 |
| 7. Participant knowledge of the interviewer | What did the participants know about the researcher? e.g. personal goals, reasons for doing the research | SG gave a presentation to participants at the start of the focus group to explain all researchers’ backgrounds and rationale for the study, 2 |
| 8. Interviewer characteristics | What characteristics were reported about the interviewer/facilitator? e.g. Bias, assumptions, reasons and interests in the research topic | SG was the chief investigator for the FERARO study – this qualitative study was nested within this trial to provide additional data on the feasibility of proceeding with a substantive clinical trial, 2 |

| **Domain 2: study design** |  |  |
| --- | --- | --- |
| *Theoretical framework* |  |  |
| 9. Methodological orientation and Theory | What methodological orientation was stated to underpin the study? e.g. grounded theory, discourse analysis, ethnography, phenomenology, content analysis | Thematic analysis, 3 |
| *Participant selection* |  |  |
| 10. Sampling | How were participants selected? e.g. purposive, convenience, consecutive, snowball | Participants were selected based on the timing of focus groups and their progress in follow-up for the FERARO study, 2 |
| 11. Method of approach | How were participants approached? e.g. face-to-face, telephone, mail, email | Mail, 2 |
| 12. Sample size | How many participants were in the study? | 13 participants, 2 |
| 13. Non-participation | How many people refused to participate or dropped out? Reasons? | 11 of those invited declined to participate, 2 |
| *Setting* |  |  |
| 14. Setting of data collection | Where was the data collected? e.g. home, clinic, workplace | Hospital, 2 |
| 15. Presence of non-participants | Was anyone else present besides the participants and researchers? | Other members of the research team to SG and DW were observers – BM, DP and MK, 2 |
| 16. Description of sample | What are the important characteristics of the sample? e.g. demographic data, date | Demographic data is given as well as timing of focus groups, 2 |
| *Data collection* |  |  |
| 17. Interview guide | Were questions, prompts, guides provided by the authors? Was it pilot tested? | 2, supplementary material |
| 18. Repeat interviews | Were repeat interviews carried out? If yes, how many? | None, 2 |
| 19. Audio/visual recording | Did the research use audio or visual recording to collect the data? | Yes, 2 |
| 20. Field notes | Were ﬁeld notes made during and/or after the interview or focus group? | None, 2 |
| 21. Duration | What was the duration of the interviews or focus group? | Approximately 2 hours for each, 2 |
| 22. Data saturation | Was data saturation discussed? | Yes, 2 |
| 23. Transcripts returned | Were transcripts returned to participants for comment and/or correction? | No, 2 |
| **Domain 3: analysis and ﬁndings** |  |  |
| *Data analysis* |  |  |
| 24. Number of data coders | How many data coders coded the data? | Two – BM and MK, 3 |
| 25. Description of the coding tree | Did authors provide a description of the coding tree? | No, 3 |
| 26. Derivation of themes | Were themes identiﬁed in advance or derived from the data? | Derived from the data, 3 |
| 27. Software | What software, if applicable, was used to manage the data? | Nvivo, 3 |
| 28. Participant checking | Did participants provide feedback on the ﬁndings? | No, 3 |
| *Reporting* |  |  |
| 29. Quotations presented | Were participant quotations presented to illustrate the themes/ﬁndings? Was each quotation identiﬁed? e.g. participant number | Yes, quotations are anonymous, 4-7 |
| 30. Data and ﬁndings consistent | Was there consistency between the data presented and the ﬁndings? | Yes, 4-7 |
| 31. Clarity of major themes | Were major themes clearly presented in the ﬁndings? | Yes, 3, figure 1 |
| 32. Clarity of minor themes | Is there a description of diverse cases or discussion of minor themes? | Yes, 3, figure 1 |
