# Supplementary material for: Facilitators and barriers to recruitment and retention in a feasibility trial of encapsulated faecal microbiota transplant to eradicate carriage of antibiotic-resistant bacteria at an academic hospital in central London: a nested qualitative study
Source: BMJ Open. 2025 Nov 19;15(11):e104783. doi: 10.1136/bmjopen-2025-104783 (PMC12636872; doi:10.1136/bmjopen-2025-104783)
Supplement: online supplemental file 2 [file bmjopen-15-11-s002.docx]

**Focus group guidance document**

Venue: Infection Seminar Room, 5^th^ floor, North Wing, St Thomas’ hospital

Anticipated number of participants: 4-8 participants

Anticipated duration: 1.5-2 hours

**Welcome & introductions (who are we, who are you)**

First, thank-you for attending this focus group to discuss your participation in the FERARO study. We are indebted to you offering up your precious time for us to gain valuable information on how to shape future research in this field. You have all been invited to participate as you have either completed the study or are in the 6-month follow-up phase. The meeting will last around 1.5-2 hours depending on how the conversations go, and how much everybody has to say.

We are here as facilitators for the discussion, we hope that for most of the time you will be doing the talking and telling us what you think about the study.

We will start by going over some ground rules, before giving an overview of the study and the reasons for conducting it as a bit of a refresher. We will then move on to asking some questions and hearing your all-important opinions!

Before we start, we want to ensure that all members are happy to be present and know that they are free to leave at any time. We hope you find the discussions interesting and engaging.

**Ground rules and overview of the session**

Because this is a group discussion, you will all hear each other’s views and opinions on this trial. We request that this discussion remains confidential within this group. For the purposes of our report, we will not be using any names to ensure anonymity.

Just to help us write a report of this study, we are recording the discussion, in addition to taking notes.

We would like everybody to have the opportunity to talk, and as we are recording, would appreciate if only one person speaks at a time.

Please remember, there are no right or wrong answers, only differing points of view. You don’t need to agree with others, but please be respectful of their opinion.

If you are required to make a telephone call or attend to other business during the meeting, we would appreciate if you could step out of the room to do this as a courtesy for other focus group members.

**Overview of the topic**

The human gut has trillions of bacteria (bugs) which are important to keep us healthy. In total these bugs are called the microbiota. The bugs are always evolving to beat antibiotics used to fight them (resistance). Resistance to antibiotics allows bugs to survive and spread. This is a growing and serious threat to worldwide health and means that doctors may be limited in the types of treatments that they can offer to patients. Without effective antibiotics even simple infections could become deadly, making routine medical procedures too dangerous to perform. There is an urgent need to find new antibiotics, but this takes time and is very expensive. There is growing interest in non-antibiotic treatments like Faecal Microbiota Transplant (FMT) to deal with this problem.

FMT is the transfer of bacteria from the guts of healthy donors (taken from their poo) into the gut of a patient. The aim is to restore a healthy balance of bacteria (reducing harmful ones and increasing good ones). It is currently used to treat patients with repeated *Clostridioides difficile* (C.diff) infection. This is an infection causing severe diarrhoea and stomach pain, normally after having antibiotics which have harmed the microbiota. FMT is very effective and safe in treating this group of patients, with success rates of over 80%. Initial research shows that it may be helpful in other conditions. Especially for getting rid of antibiotic-resistant bacteria (ARB) found in some patients' guts.

This study is looking at whether giving FMT to patients with ARB is an achievable treatment. We will see if it is both safe and acceptable to patients, without side effects. This will allow doctors to treat infections in these patients better. If the treatment works, it could be rapidly brought into the NHS. This could help patients who have ARB and can't be treated with current antibiotics. Unfortunately, at this stage we do not have any study results to share with you but hope to be able to when they become available in the future.

**Introductions**

To get to know each other a little bit, we would like you to introduce yourself to the group, where you have travelled from today and how your journey to the hospital was.

***Facilitator to start***

Thank everybody for their introductory statement.

**Topics for discussion**

We will now go on to several scripted questions. There will also be an opportunity at the end to make comments outside of those asked.

***Note the generic discussion probes at the end of the section***

Could you tell us what made you participate in the trial?

Specific probes:

- What are your thoughts on antimicrobial resistance?
- How big a problem do you see it as?
- Do you think we (medical profession, politicians, policy makers etc.) are doing enough to tackle it?

Could you describe any health benefits you have encountered by participating in this trial?

Specific probes:

- Health benefit = something that has a positive effect on your health
- Health is defined as a state of complete physical, mental and social well-being, and not merely the absence of disease or infirmity (WHO, 1948)

Could you tell us about any challenges you encountered with this trial?

Specific probes:

- Challenges might include, but are no means limited to, stool or blood sampling including sending in the samples via post, face-to-face study visits, travel to the hospital, COVID-19 pandemic
- Could you tell us about how you overcame these challenges?

Could you tell us what went well during your time participating in the trial?

Specific probes:

- Please could you expand on your personal beneficial experience?
- Can you tell us how we could enhance or improve your trial experience?
- What further support, or help, would make this trial more efficient?

We would like to ask for your opinion on the acceptability of faecal microbiota transplantation (FMT) as a treatment

Specific probes:

- Would having a synthetically produced consortia of bacteria be more appealing?
- What are your thoughts on taking capsules vs. other ways we administer FMT (via nasojejunal tube or colonoscopy)
- What were your thoughts on receiving a drug manufactured from faeces?
- Did the dosing regimen (3 consecutive days) suit you? Is there another dosing regimen that would have been more convenient?

Generic discussion probes:

- Please could you tell me more about/ expand on that?
- What do you mean by that?
- Is there anything else you would like to mention?

**Closure of discussion**

In your opinion, did we discuss all the relevant issues?

Would you like to add anything else to your responses?

Would you like to discuss any other points related to the trial?

Finally, of the things discussed today, which to you was the most important?

**Summary**

We will now bring the focus group to a close.

***Summarise briefly what has been discussed***

We will be providing you follow-up information both on the results of this focus group meeting and of the trial itself once they are published.

**End**

We wish you all a safe journey home and thank-you again for your participation today.
