# Supplementary material for: Facilitators and barriers to recruitment and retention in a feasibility trial of encapsulated faecal microbiota transplant to eradicate carriage of antibiotic-resistant bacteria at an academic hospital in central London: a nested qualitative study
Source: BMJ Open. 2025 Nov 19;15(11):e104783. doi: 10.1136/bmjopen-2025-104783 (PMC12636872; doi:10.1136/bmjopen-2025-104783)
Supplement: Supplementary file 3 [file bmjopen-2025-104783.reviewer_comments.pdf]

PEER REVIEW HISTORY

BMJ Open publishes all reviews undertaken for accepted manuscripts. Reviewers are asked to complete a checklist review form and are provided with free text boxes to elaborate on their assessment. These free text comments are reproduced below.

ARTICLE DETAILS

Title (Provisional)

Facilitators and barriers to recruitment and retention in a feasibility trial of encapsulated faecal microbiota transplant to eradicate carriage of antibiotic-resistant bacteria at an academic hospital in central London: a nested qualitative study

Authors

Merrick, Blair; Prossomariti, Désirée; Kertanegara, Michael; Wyatt, David; Goldenberg, Simon

---

VERSION 1 - REVIEW

Reviewer 1

Name zhang, faming

Affiliation the Second Affiliated Hospital of Nanjing Medical University, Medical Center for Digestive Diseases

Date 31-May-2025

COI None

This is a very interesting study. I strongly recommend conducting such investigations and research. However, I have major comments that warrant the authors' attention.

Comment 1: The perception bias toward FMT among physicians and patients largely depends on how FMT is implemented. Crude FMT cannot overcome its inherent limitations, regardless of how attractively it is named. FMT providers or researchers should transparently disclose their methodology, sources, composition, etc., to gain broader acceptance. The recently published PRIM 2024 guidelines (preferred reporting items for microbiotherapy checklist, J Gastroenterol Hepatol. 2025) were developed precisely to address these issues, providing detailed reporting standards. I suggest the authors to reference and discuss these guidelines.

Comment 2:

The authors' description of the current landscape of FMT applications—particularly in countries other than the UK—is notably limited. BMJ Open is an international journal, and published research should reflect global progress.

Comment 3:

Similar studies have been reported in other journals. The authors should clarify how their work distinguishes itself from prior research and what novel questions it resolves.

**Reviewer**	**2**
**Name**	**O'Brien, Alastair**
**Affiliation**	**UCL, Division of Medicine**
**Date**	**23-Jul-2025**
**COI**	**None**

This nested qualitative study within the FERARO single centre clinical trial that aimed to identify facilitators and barriers to the delivery of an encapsulated faecal microbiota transplant (FMT).

I have a few comments that predominantly relate to producing a more focused manuscript that identifies their key findings succinctly and describes how these could be used to improve participation within future FMT trials. The study has identified several interesting and informative participant responses and the concluding paragraph is well written but much of the other text is too lengthy and lacks focus.

The introduction is too long. The first 2 paragraphs describing AMR and FMT could be shortened substantially. Equally it was uncertain for the rationale to include the 3rd paragraph - was this the inspiration for this nested study - if so this should be explicitly stated.

The results section is very long. I am not a qualitative researcher but wonder if this section could be shortened substantially. The text contains a large amount that has been directly lifted from the participants responses rather than analyses of the interviews. Perhaps a description of the major findings from each section could be included and the individual responses put in a supplementary results section.

Again the discussion is very long. The first paragraph does not really focus on the results at all. The concluding paragraph is very good and perhaps much of this could be moved to the first paragraph with the discussion based around how the team believe they could incorporate their learnings and what are the next practical steps to achieve this.

VERSION 1 - AUTHOR RESPONSE

Reviewer: 1

Comments to the Author:
This is a very interesting study. I strongly recommend conducting such investigations and research. However, I have major comments that warrant the authors' attention.
We thank the reviewer for their kind assessment of our research.

Comment 1: The perception bias toward FMT among physicians and patients largely depends on how FMT is implemented. Crude FMT cannot overcome its inherent limitations, regardless of how attractively it is named. FMT providers or researchers should transparently disclose their methodology, sources, composition, etc., to gain broader acceptance. The recently published PRIM 2024 guidelines (preferred reporting items for microbiotherapy checklist, J Gastroenterol Hepatol. 2025) were developed precisely to address these issues, providing detailed reporting standards. I suggest the authors to reference and discuss these guidelines.
We thank the reviewer for highlighting this paper; however, we do not feel that it is relevant for a qualitative study of patient participants. We have reported these items previously in the FERARO trial manuscript (https://doi.org/10.1016/j.jinf.2025.106504) and publications regarding preparation of the lyophilised encapsulated FMT (https://doi.org/10.1016/j.ijpharm.2025.125703 and https://doi.org/10.1016/j.jconrel.2022.08.012). We have discussed the transparency issue with FMT and the participant perceptions of FMT within the manuscript.

Comment 2:
The authors' description of the current landscape of FMT applications—particularly in countries other than the UK—is notably limited. BMJ Open is an international journal, and published research should reflect global progress.
We do not believe it is within the scope of this paper to describe the landscape of FMT globally – there are numerous review papers that already do this. If we attempted to do this it would further lengthen the manuscript, which both the editor and reviewer 2 were keen to shorten. We have referenced to several national and international guidelines regarding the use of FMT for recurrent *C. difficile* infection.

Comment 3:
Similar studies have been reported in other journals. The authors should clarify how their work distinguishes itself from prior research and what novel questions it resolves.
We have only identified one other similar study (https://doi.org/10.1136/bmjopen-2020-039471), but this was for a different disease process. Our work distinguishes itself by informing about the delivery of a larger substantive RCT in gastrointestinal MDRO eradication and the use of a lyophilised encapsulated form of FMT. It also informs regarding the delivery of FMT studies more widely including challenges around dosing visits, dosing frequency, and the return of study samples from remote visits.

Reviewer: 2

Comments to the Author:
This nested qualitative study within the FERARO single centre clinical trial that aimed to identify facilitators and barriers to the delivery of an encapsulated faecal microbiota transplant (FMT).
I have a few comments that predominantly relate to producing a more focused manuscript that identifies their key findings succinctly and describes how these could be used to improve participation within future FMT trials. The study has identified several interesting and informative participant responses and the concluding paragraph is well written but much of the other text is too lengthy and lacks focus.
We thank the reviewer for their kind assessment of our paper and highlighting areas in which our manuscript could be improved, we have made some changes, described in greater detail below. Most

notably in reducing the total word count from over 7,000 to under 5,000, we hope that you find the revised manuscript more focused.

The introduction is too long. The first 2 paragraphs describing AMR and FMT could be shortened substantially. Equally it was uncertain for the rationale to include the 3rd paragraph - was this the inspiration for this nested study - if so this should be explicitly stated.
The first 2 paragraphs have been shortened by over 100 words (approx. 40%). We agree that the detail included in the 3rd paragraph is unnecessary. The study described was not the inspiration for our study and have removed the paragraph.

The results section is very long. I am not a qualitative researcher but wonder if this section could be shortened substantially. The text contains a large amount that has been directly lifted from the participants responses rather than analyses of the interviews. Perhaps a description of the major findings from each section could be included and the individual responses put in a supplementary results section.
We agree that the results section is long and have substantially shortened it. To avoid the need to refer to a separate file we have retained quotes within the main body of the text. We have shortened or removed many of these as well as the accompanying text to reduce the overall word count.

Again the discussion is very long. The first paragraph does not really focus on the results at all. The concluding paragraph is very good and perhaps much of this could be moved to the first paragraph with the discussion based around how the team believe they could incorporate their learnings and what are the next practical steps to achieve this.
The first paragraph has been shorted, as well as the entire discussion (almost 50% shorter). Practical steps are referred to throughout including redesign of capsules, AMR promotion, videos or multimedia participant facing materials, home FMT dosing, and reducing participant in-person visits in future trials.

VERSION 2 - REVIEW

**Reviewer**	**2**
**Name**	**O'Brien, Alastair**
**Affiliation**	**UCL, Division of Medicine**
**Date**	**01-Oct-2025**
**COI**	

The authors have addressed my comments well. I enjoyed the paper.
